# *TP53* Mutation Status and Biopsy Lesion Type Determine the Immunotherapeutic Stratification in Non-Small-Cell Lung Cancer

**DOI:** 10.3389/fimmu.2021.732125

**Published:** 2021-09-17

**Authors:** Jun Lu, Runbo Zhong, Yuqing Lou, Minjuan Hu, Zhengyu Yang, Yanan Wang, Ya Chen, Benkun Zou, Wei Zhang, Huimin Wang, Baohui Han

**Affiliations:** ^1^Department of Pulmonary Medicine, Shanghai Chest Hospital, Shanghai Jiao Tong University, Shanghai, China; ^2^Shanghai Institute of Thoracic Oncology, Shanghai Chest Hospital, Shanghai Jiao Tong University, Shanghai, China; ^3^Translational Medical Research Platform for Thoracic Oncology, Shanghai Chest Hospital, Shanghai Jiao Tong University, Shanghai, China

**Keywords:** immunotherapy, biomarker, *TP53* mutation, source of tissue, non-small-cell lung cancer

## Abstract

Immunotherapy, a chemotherapy-free process, has emerged as a promising therapeutic strategy to prolong the overall survival (OS) of patients with non-small-cell lung cancer (NSCLC). However, effective stratification factors for immunotherapy remain unclear. The purpose of this study was to discuss the potential stratification factors of NSCLC immunotherapy using immune checkpoint inhibitors (ICIs) by integrating genomic profiling and tumor lesion–type information. In this study, 344 patients with NSCLC, whose clinical and tissue (including metastatic and primary lesions) mutation information was available, were included. The potential gene mutation status for predicting the outcomes of immunotherapy was screened by comparing the difference in mutation frequency between responders and non-responders. Our results indicated that the potential predictors of immunotherapy were significantly different, especially between patients with *TP53*(+) (including metastatic and primary lesions) and *TP53*(−) (including metastatic and primary lesions). According to this classification, patients with NSCLC who suggested immunotherapy had a higher OS than those who did not (25 months *vs.* 7 months, *P* < 0.0001, hazard ratio = 0.39). Collectively, this study provides a new perspective for screening immunotherapy predictors in NSCLC, suggesting that the *TP53* mutation status and source of biopsy tissue should be considered during the development of immunotherapy biomarkers.

## Introduction

Non-small-cell lung cancer (NSCLC) is one of the most malignant diseases, accounting for approximately 85% of lung cancer (1–3). Chemotherapy has played an important role in NSCLC treatment (4–6). Since 2009, targeting the tyrosine kinase inhibitors (TKIs) has changed the clinical course for NSCLC patients harboring epidermal growth factor receptor (EGFR) mutations and anaplastic lymphoma kinase (ALK) and proto-oncogene receptor tyrosine kinase (ROS1) rearrangements (7–10). However, for patients without driver gene mutations, the therapeutic regimen remains limited (6). Fortunately, recent advances in immunotherapy have provided new therapeutic targets for lung cancer (4, 11–13).

Immunity checkpoint inhibitors (ICIs), including programmed cell death protein 1 (PD1) and programmed cell death protein 1 ligand 1 (PD-L1) inhibitors, block the PD1/PD-L1 signaling pathway, relieve the immune escape of tumor cells, and kill tumor cells by activating cytotoxic T cells (14–16). Several clinical trials have reported that immunotherapy can significantly improve the overall survival (OS) of patients with NSCLC at first, second, and third lines (4, 11, 17–19). However, some patients in these trials received a long-term OS benefit, whereas others received a short-term OS benefit although all patients were characterized by similar pathological types and received the same ICI (20). These findings signify the urgent need to identify effective stratification factors for immunotherapy.

Several biomarkers, including PD-L1 expression, tumor mutation burden (TMB), and microsatellite instability (MSI), have been developed to distinguish responders to immunotherapy from non-responders in NSCLC (20–25). Among these biomarkers, PD-L1 expression and TMB have been included in National Comprehensive Cancer Network (NCCN) guideline for guiding immunotherapeutic clinical practice (6). However, the above biomarkers are associated with certain limitations (not all patients with a high PD-L1 expression/TMB/MSI responded well to immunotherapy), indicating that biomarker development needs to be explored further (26, 27). In the present study, we screened the immunotherapy stratifying factors through the classification of *TP53* mutation status and biopsy lesion type in 344 patients with NSCLC who received immunotherapy.

## Materials and Methods

### Patients and Samples

This study enrolled 344 NSCLC patients who were approved by the institutional review board of the Memorial Sloan-Kettering Cancer Center (MSKCC) (26). All patients with NSCLC had received at least one cycle of immunotherapy (ICIs such as nivolumab, atezolizumab, ipilimumab, pembrolizumab, avelumab, tremelimumab, and durvalumab). All enrolled patients with NSCLC signed the informed consent for the companion study. Among the 344 patients with NSCLC, we obtained metastatic lesion samples from 176 patients and primary lesion samples from 168 patients. In addition, 217 patients harbored *TP53* mutations, and 127 patients did not have this mutation.

### Sequencing

The sequencing methods used in the study have been described in detail previously (28). Briefly, DNA was extracted from metastatic and primary lesions, end-repaired, adapter-ligated, and amplified. The quality control for amplified products was performed, following which they were sequenced. The MSK-IMPACT panel was used for targeted sequencing. Somatic tumor mutation calling was performed between the tissue sequencing and white blood cell (WBC) sequencing data. All somatic tumor mutation data and clinical information were downloaded from the cBioPortal for Cancer Genomics (www.cbioportal.org).

### Mutation Frequency Analysis

The mutation frequency for the top 30 genes for all 344 patients with NSCLC was calculated. The most significant differences in mutation genes were screened by comparing the mutation frequency between patients with OS >12 months and those with OS ≤12 months. Here, the patients who received immunotherapy with OS >12 months were defined as “responder”; the patients who received immunotherapy with OS ≤12 months were defined as “non-responders”. Furthermore, the mutation frequency between different subgroups was analyzed using samples from metastatic and primary lesions; *TP53*(+) and *TP53*(−) patients; *TP53*(+) patients with metastatic lesions; *TP53*(+) patients with primary lesions; *TP53*(−) patients with metastatic lesions; and *TP53*(−) patients with primary lesions.

### OS Analysis

The OS analysis was performed according to the methods described in our previous studies (5, 29, 30). We compared the mutation frequencies between different subgroups to select different predictors for stratification. GraphPad Prism 5 software was used to calculate the differences between different subgroups. The log-rank test was used to analyze significant differences (*P* values) between different cohorts. Hazard ratios (HRs) were calculated for OS.

### Statistical Analysis

The log-rank (Mantel-Cox) test was used to test the difference of survival time between different cohorts. In addition, HRs and exact 95% confidence intervals (CIs) were reported. Differences were considered significant at ^*^ *p* < 0.05, ^**^ *p* < 0.01, and ^***^ *p* < 0.001.

## Results

### The Mutational Differences Between Responders and Non-Responders Potentially Be Used as Predictor in NSCLC Immunotherapy

**Figure 1:**
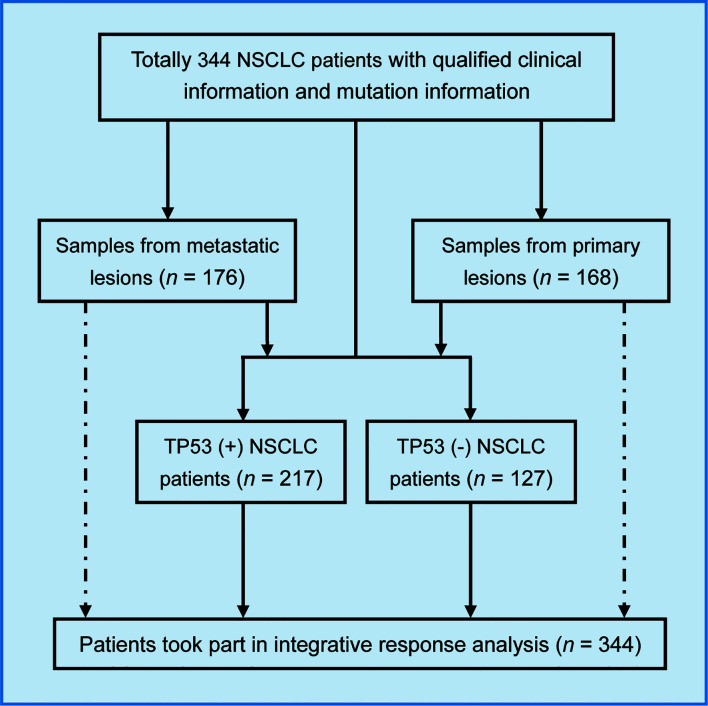
Flow chart showing patient selection and analysis method used in the study.

**Figure 2:**
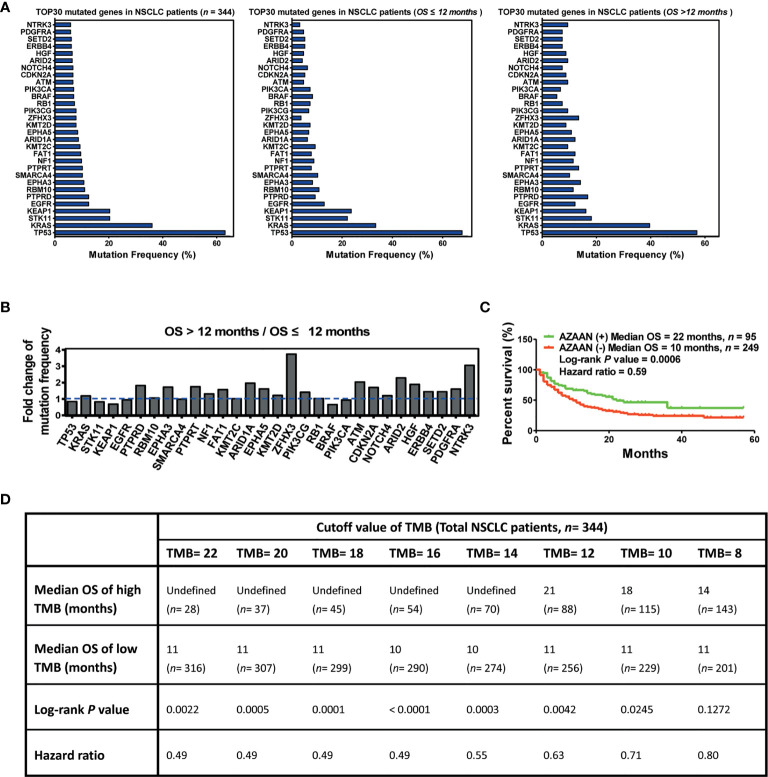
AZAAN mutation status as a stratifying predictor of immunotherapy in NSCLC. **(A)** Left: Mutation frequency of the top 30 genes. Middle: Mutation frequency of the top 30 genes for patients with NSCLC having OS ≤12 months. Right: Mutation frequency of the top 30 genes for patients with NSCLC having OS >12 months. **(B)** Fold change in mutation frequency with OS >12 months/OS ≤12 months for the top 30 genes. **(C)** Kaplan–Meier curve analysis of OS stratification using the *AZAAN* (*ARID1A*, *ZFHX3*, *ATM*, *ARID2*, and *NTRK3*) mutation status. **(D)** Different TMB cutoffs used as a predictor for immunotherapy.

In this study, 344 patients with NSCLC (with clinical and mutation information) were screened to identify immunotherapy predictors, from an MSKCC cohort (1,661 cancer patients including NSCLC, melanoma, glioma, and colorectal cancer) (**Figure 1**). As shown in **Figure 2A**, in the present cohort, patients harboring *TP53* mutations accounted for more than 60% of cases, followed by *KRAS*, *STK11*, and *KEAP1*. Next, the top 30 genes with mutation frequency were selected for further analysis. Our results indicated a significant difference in the mutation frequency between patients with OS ≤12 months and those with OS >12 months. Next, we calculated the ratios of mutation frequency for the top 30 genes and obtained an altered trend chart (**Figure 2B**). The top five altered genes, namely, *ARID1A*, *ZFHX3*, *ATM*, *ARID2*, and *NTRK3*, were named AZAAN. Therefore, we evaluated the effect of the predictor-AZAAN on responsive stratification in patients who had received immunotherapy. The results indicated that patients harboring AZAAN(+) received more OS benefits from immunotherapy than those patients harboring AZAAN(−) [AZAAN(+) *vs.* AZAAN(−): 22 months *vs.* 10 months, log-rank *P* value = 0.0006, HR = 0.59] (**Figure 2C**). TMB can be used as a predictor for immunotherapy response. As shown in **Figure 2D**, the log-rank *P*-value and HR of the predictor TMB (TMB ≥ 14) were superior to those of the predictor-AZAAN. However, either the predictor TMB or the predictor AZAAN just identified a small proportion of patients (no more than 28%) who were suggested to receive immunotherapy, indicating that immunotherapy predictors of NSCLC need to be further explored.

### Mutation Profiling From Different Biopsy Lesions Determine the Predictor Screening

**Figure 3:**
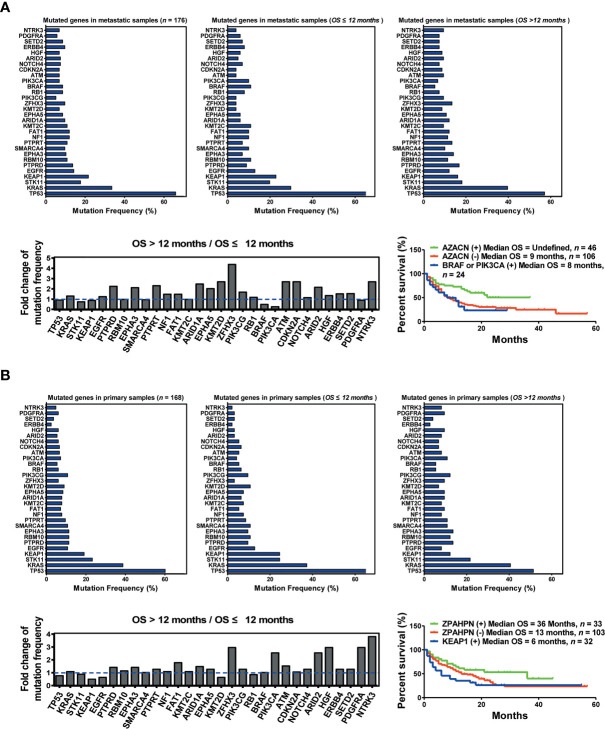
Biopsy lesion type affects the stratifying factors of immunotherapy. **(A)** Up left: Mutation frequency of the top 30 genes in the metastatic sample cohort. Upper middle: Mutation frequency of the top 30 genes in the metastatic sample cohort with OS ≤12 months. Upper right: Mutation frequency of the top 30 genes in the metastatic sample cohort with OS >12 months. Down left: Fold change in mutation frequency with OS >12 months/OS ≤12 months for the top 30 genes. Down right: Kaplan–Meier curve analysis of OS stratification using the AZACN (*ARID1A*, *ZFHX3*, *ATM*, *CDKN2A*, and *NTRK3*) mutation status. **(B)** Upper left: Mutation frequency of the top 30 genes in the primary sample cohort. Upper middle: Mutation frequency of the top 30 genes in the primary sample cohort with OS ≤12 months. Upper right: Mutation frequency of the top 30 genes in the primary sample cohort with OS >12 months. Down left: Fold change in mutation frequency with OS >12 months/OS ≤12 months for the top 30 genes. Down right: Kaplan–Meier curve analysis of OS stratification using the *ZPAHPN* (*ZFHX3*, *PIK3CA*, *ARID2*, *HGF*, *PDGFRA*, and *NTRK3*) mutation status.

To further understand the differences between primary and metastatic lesions, we divided 334 patients into two cohorts, namely, primary and metastatic sample cohorts. A comparison of two cohorts revealed that the mutation frequencies of the top 30 genes were significantly different between them. In addition, the mutation frequency of multiple genes changed remarkably between the OS >12 months cohort and the OS ≤12 months cohort in the metastatic sample cohort. The top five upregulated genes (AZACN: *ARID1A*, *ZFHX3*, *ATM*, *CDKN2A*, and *NTRK3*) and the bottom two downregulated genes (*BRAF* and *PIK3CA*) were selected as combined predictors for screening responders from non-responders. The results suggested that patients harboring AZACN(+) received more OS benefits from immunotherapy than those harboring AZACN(−) or harboring *BRAF* and *PIK3CA* (+) [AZACN(+) *vs.* AZACN(−) *vs. BRAF* and *PIK3CA* (+) = undefined *vs.* 9 months *vs.* 8 months] (**Figure 3A**). In the primary sample cohort, the top six upregulated genes (ZPAHPN: *ZFHX3*, *PIK3CA*, *ARID2*, *HGF*, *PDGFRA*, and *NTRK3*) and the bottom downregulated gene (*KEAP1*) were selected as combined predictors for screening responders from non-responders. The results indicated that patients harboring ZPAHPN(+) received more OS benefits than those harboring ZPAHAN(−) or *KEAP1*(+) [ZPAHPN(+) *vs.* ZPAHAN(−) *vs. KEAP1*(+) = 36 months *vs.* 13 months *vs.* 6 months] (**Figure 3B**). These results suggest that biopsy lesion type potentially affects biomarker screening for immunotherapy.

### Effect of Biopsy Lesion Types on Predictor Development in the *TP53*(+) Patients

**Figure 4:**
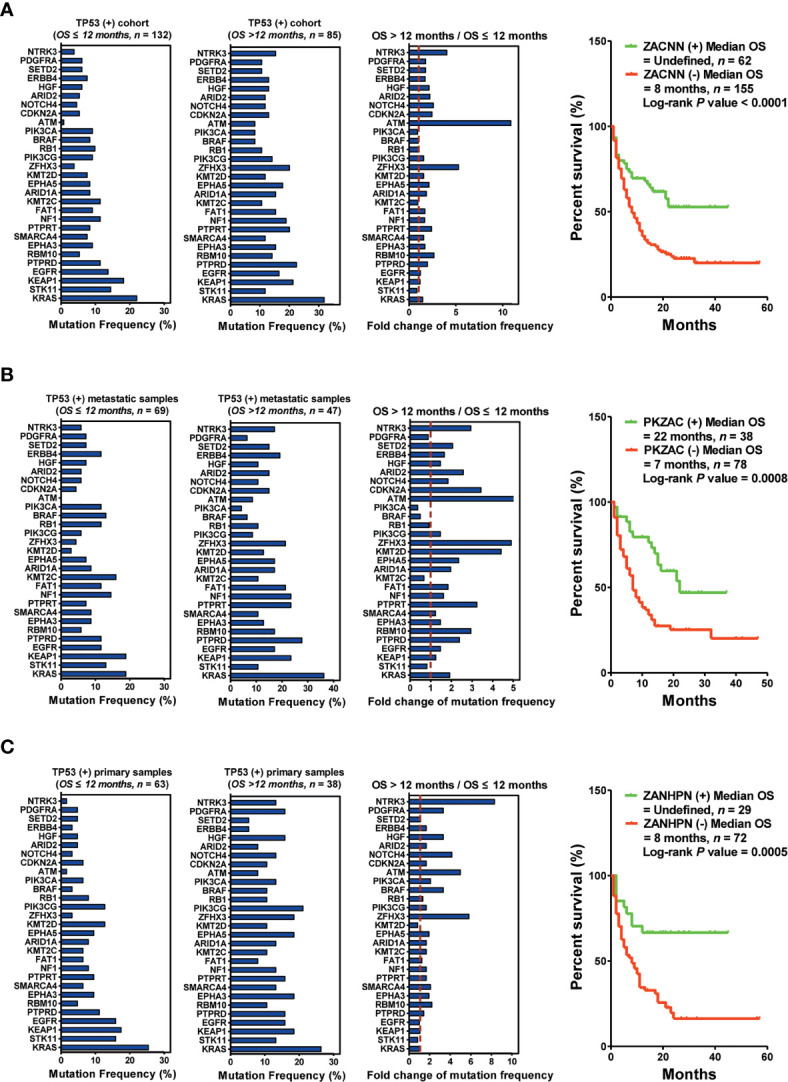
Biopsy lesion type affects the stratifying factors of immunotherapy in patients harboring *TP53*(+) mutation. **(A)** Left: Mutation frequency of the top 30 genes in the *TP53*(+) cohort with OS ≤12 months. Mutation frequency of the top 30 genes in the *TP53*(+) cohort with OS >12 months. Fold change in mutation frequency with OS >12 months/OS ≤12 months for the top 30 genes. Right: Kaplan–Meier curve analysis of OS stratification using the *ZACNN* (*ZFHX3*, *ATM*, *CDKN2A*, *NOTCH4*, and *NTRK3*) mutation status. **(B)** Left: Mutation frequency of the top 30 genes in the *TP53*(+) metastatic sample cohort with OS ≤12 months. Mutation frequency of the top 30 genes in the *TP53*(+) metastatic sample cohort with OS >12 months. Fold change in mutation frequency with OS >12 months/OS ≤12 months for the top 30 genes. Right: Kaplan–Meier curve analysis of OS stratification using the *PKZAC* (*PTPRT*, *KMT2D*, *ZFHX3*, *ATM*, and *CDKN2A*) mutation status. **(C)** Left: Mutation frequency of the top 30 genes in the *TP53*(+) primary sample cohort with OS ≤12 months. Mutation frequency of the top 30 genes in the *TP53*(+) primary sample cohort with OS >12 months. Fold change in mutation frequency with OS >12 months/OS ≤12 months for the top 30 genes. Right: Kaplan–Meier curve analysis of OS stratification using the *ZANHPN* (*ZFHX3*, *ATM*, *NOTCH4*, *HGF*, *PDGFRA*, and *NTRK3*) mutation status.

To precisely screen the potential responders of immunotherapy *via* DNA profiling, we performed an integrated analysis based on *TP53* mutation status as well as the biopsy lesion type. We found a significant difference in the mutation frequency of the top 30 genes between patients harboring *TP53*(+) and those harboring *TP53*(−). For patients harboring *TP53*(+), the top five upregulated genes (ZACNN: *ZFHX3*, *ATM*, *CDKN2A*, *NOTCH4*, and *NTRK3*) were selected as predictors for screening responders from non-responders. The results indicated that patients harboring ZACNN(+) received more OS benefits from immunotherapy than those harboring ZACNN(−) [ZACNN(+) *vs.* ZACNN(−) = undefined *vs.* 8 months, *P* < 0.0001] (**Figure 4A**). Using this stratification method, about 28.6% of *TP53*(+) patients were screened for immunotherapy recommendation. Furthermore, 217 patients harboring *TP53*(+) were divided into two cohorts according to the biopsy lesion type (metastatic sample and primary sample cohorts). In the metastatic sample cohort (116 patients), the top five upregulated genes (PKZAC: *PTPRT*, *KMT2D*, *ZFHX3*, *ATM*, and *CDKN2A*) were selected as predictors to screen the responders. Patients harboring PKZAC(+) received more OS benefits from immunotherapy than those harboring PKZAC(−) [PKZAC(+) *vs.* PKZAC(−) = 22 months *vs.* 7 months, *P* = 0.0008] (**Figure 4B**). In the primary sample cohort (101 patients), the top six upregulated genes (ZANHPN: *ZFHX3*, *ATM*, *NOTCH4*, *HGF*, *PDGFRA*, and *NTRK3*) were selected as predictors for screening responders from non-responders. The patients harboring ZANHPN(+) received more OS benefits from immunotherapy than those harboring ZANHPN(−) [ZANHPN(+) *vs.* ZANHPN(−) = 29 months *vs.* 8 months, *P* = 0.0005] (**Figure 4C**).

### Effect of Biopsy Lesion Types on Predictor Development in the *TP53*(−) Patients

**Figure 5:**
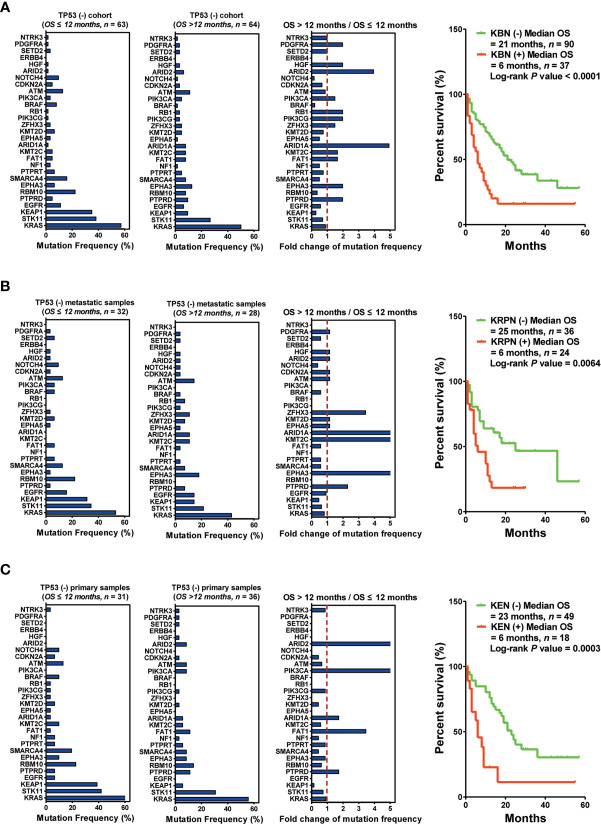
Biopsy lesion type affects the stratifying factors of immunotherapy in patients without *TP53*(+) mutation. **(A)** Left: Mutation frequency of the top 30 genes in the *TP53*(−) cohort with OS ≤12 months. Mutation frequency of the top 30 genes in the *TP53*(−) cohort with OS >12 months. Fold change in mutation frequency with OS >12 months/OS ≤12 months for the top 30 genes. Right: Kaplan–Meier curve analysis of OS stratification using the *KBN* (*KEAP1*, *BRAF*, and *NOTCH4*) mutation status. **(B)** Left: Mutation frequency of the top 30 genes in the *TP53*(−) metastatic sample cohort with OS ≤12 months. Mutation frequency of the top 30 genes in the *TP53*(−) metastatic sample cohort with OS >12 months. Fold change in mutation frequency with OS >12 months/OS ≤12 months for the top 30 genes. Right: Kaplan–Meier curve analysis of OS stratification using the *KRPN* (*KEAP1*, *RBM10*, *PIK3CA*, and *NOTCH4*) mutation status. **(C)** Left: Mutation frequency of the top 30 genes in the *TP53*(−) primary sample cohort with OS ≤12 months. Mutation frequency of the top 30 genes in the *TP53*(−) primary sample cohort with OS >12 months. Fold change in mutation frequency with OS >12 months/OS ≤12 months for the top 30 genes. Right: Kaplan–Meier curve analysis of OS stratification using the *KEN* (*KEAP1*, *EGFR*, and *NOTCH4*) mutation status.

Next, 127 patients without *TP53* mutations were subjected to another set of analyses. The bottom three downregulated genes (KBN: *KEAP1*, *BRAF*, and *NOTCH4*) were selected as predictors for screening responders from non-responders. The results indicated that the patients harboring KBN(−) received more OS benefits from immunotherapy than those harboring KBN(+) [KBN(−) vs. KBN(+) = 21 months vs. 6 months, *P* < 0.0001] (**Figure 5A**). In the metastatic sample cohort (60 patients), the bottom four downregulated genes (KRPN: *KEAP1*, *RBM10*, *PIK3CA*, and *NOTCH4*) were selected as predictors for screening responders from non-responders. Patients harboring KRPN(−) received more OS benefits from immunotherapy than those harboring KRPN(+) [KRPN(−) vs. KRPN(+) = 26 months vs. 6 months, *P* = 0.0064] (**Figure 5B**). In the primary sample cohort (67 patients), the bottom three downregulated genes (KEN: *KEAP1*, *EGFR*, and *NOTCH4*) were selected as predictors for screening responders from non-responders. Patients harboring KEN(−) received more OS benefits from immunotherapy than those harboring KEN(+) [KEN(−) vs. KEN(+) = 23 months vs. 6 months, *P* = 0.0003] (**Figure 5C**).

### Integration of *TP53* Mutation Status and Biopsy Lesion Types for Predictor Development in Immunotherapy

**Figure 6:**
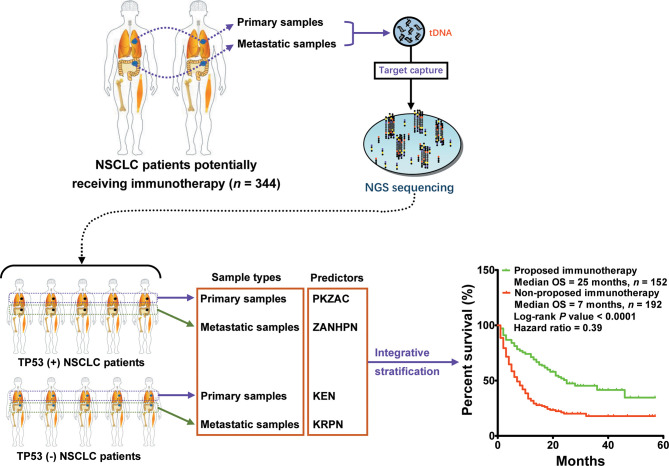
Integrative stratification using different *TP53* mutation status and biopsy lesion types for immunotherapy in NSCLC. Samples from different lesions (metastatic and primary lesions) were subjected to standard high-throughput sequencing. The mutation profile of each patient was used for driver gene-based subtype analysis. In total, 344 patients with NSCLC were divided into two cohorts: [*TP53*(+) cohort and *TP53*(−) cohort]. According to biopsy lesion type, NSCLC patients were further divided into *TP53*(+) metastatic sample cohort, *TP53*(+) primary sample cohort, *TP53*(−) metastatic sample cohort, and *TP53*(−) primary sample cohort. Each cohort developed an independent optimal predictor. Patients who potentially received more OS benefits from immunotherapy were screened by integrative stratification.

Here, we observed an interesting phenomenon, that is, the predictors derived from *TP53*(+) patients were commonly used to screen responders, whereas those derived from *TP53*(−) patients were used to screen non-responders. Collectively, we performed a multiple classification analysis on 217 patients with *TP53*(−) and 127 patients with *TP53*(+), as well as the source of tissue, and identified four predictors (PKZAC, ZANHPN, KEN, and KRPN). Next, we provided stratifying management for patients receiving immunotherapy. Among the 344 patients with NSCLC, 152 patients were proposed to receive immunotherapy with a median OS of 25 months, and 192 patients were proposed not to receive immunotherapy with a median OS of 7 months (*P* < 0.0001, HR = 0.39) (**Figure 6**). Approximately 44.2% of patients were recommended to receive immunotherapy, with a reduced death risk of 61%. Collectively, the *TP53* mutation status and biopsy lesion type potentially determined the stratifying pattern of immunotherapy.

## Discussion

Immunotherapy is a novel therapeutic regimen that functions by blocking the PD1/PD-L1 signaling pathway, relieving the immune escape of tumor cells, and activating cytotoxic T cells. It has been demonstrated to play a critical role in NSCLC treatment (4, 6, 11–13). However, the effective stratifying factors for immunotherapy remain unclear. In the present study, 344 patients with NSCLC, whose clinical and mutation information was available, were enrolled to screen potential stratifying factors for immunotherapy.

Patients with a high PD-L1 expression in tumor tissue received more OS benefits from immunotherapy (22). This theory is beyond reproach because the immune escape of tumor cells is based on the activation of the PD1/PD-L1 signaling pathway (13, 14). The patients harboring higher expression of PD-L1 causes a greater response to ICIs. Based on the PD-L1 predictor, multiple important clinical trials of ICIs have achieved the OS endpoint (4, 11, 17). Therefore, PD-L1 plays a pioneering role in promoting the clinical practice of immunotherapy (22). Nevertheless, further studies found that not all patients with a high PD-L1 expression responded well to immunotherapy, and not all patients without PD-L1 expression not responded to immunotherapy (31–34). This phenomenon has motivated the researchers to screen new predictors that can be used for clinical stratification of immunotherapy. In 2015, Rizvi et al. first proposed that tumor mutation load could potentially be used for stratification of immunotherapy in NSCLC (24). They believed that numerous somatic mutations encoded multiple neoantigens, which determined the response of patients to ICIs (24). The predictor TMB was demonstrated to be effective in several subsequent studies (21, 31, 35). However, similar to PD-L1, not all patients with a high TMB showed a good response to immunotherapy or not all patients with low or moderate TMB responded to immunotherapy (31, 36, 37). These findings led the researchers to believe that TMB is not an enough effective predictor for immunotherapy (27). In addition, MSI can be regarded as a candidate predictor for stratification of immunotherapy (25). Altogether, the above predictors (PD-L1, TMB, and MSI) play an important role in the development of immunotherapy.

We found that the predictors (PD-L1, TMB, and MSI) were independent of *TP53* mutation status and the source of biopsy tissue. Based on existing evidence, there may be great differences in tumor biology between patients with NSCLC harboring *TP53* mutations and those without *TP53* mutations, and the mutation profiling of metastatic lesions may differ from that of primary lesions (38–43). In the present study, we found that the predictor AZAAN potentially guided the stratification of immunotherapy, regardless of the tissue source and *TP53* mutation status. These results suggest that a combination of mutated genes can potentially be used as a predictor for immunotherapy by comparing the mutation frequency between responders and non-responders. However, the mutation landscape of metastatic lesions is different from that of primary lesions. Whether these differences determine the response rate to immunotherapy remains unclear. Therefore, we subdivided the 344 patients’ cohort into two cohorts (metastatic sample cohort and primary sample cohort) according to the source of biopsy tissue and performed predictor screening analysis. Interestingly, the results demonstrated a significant difference in predictors between the metastatic and primary sample cohorts. These results indicate that the biopsy lesion type should be considered during mutation profiling analysis to screen the predictors of immunotherapy.

Based on the mutational difference between metastatic and primary lesions, as well as the *TP53*-affected tumor biology difference, whether the *TP53* mutation status combined with the biopsy lesion type is associated with the predictor of immunotherapy remains unclear. Previous studies have shown a higher *TP53* mutation frequency in metastatic lesions than in primary lesions, and patients harboring *TP53* mutations potentially receiving more OS benefits from immunotherapy (41). In the present cohort, more than 60% of patients with NSCLC harbored *TP53* mutations. Among these patients, the metastatic and primary sample cohorts were included. After predictor screening, we found that the predictor of PKZAC for *TP53*(+) metastatic sample cohort and the predictor of ZANHPN for *TP53*(+) primary sample cohort could potentially be used for stratification of immunotherapy. For the *TP5*3(−) cohort, the predictor changed to KRPN in the *TP53*(−) metastatic sample cohort and KEN in the *TP53*(−) primary sample cohort. These results indicate that the optimal predictor differs according to *TP53* mutation status and biopsy lesion type. In addition, previous studies reported that patients harboring *KEAP1* or *STK11* mutations received shorter OS benefits from immunotherapy (44, 45). Our results provide a new perspective on this issue. We did not observe a difference in the OS for patients harboring *TP53* mutations, regardless of *KEAP1* and *STK11* mutations, after receiving immunotherapy. If the *TP53*(−) patients harbor *KEAP1* and *STK11* mutations, the OS is remarkably shorter than those patients without *KEAP1* and *STK11* mutations, after receiving immunotherapy. One of the limitations of the study was the small sample size, especially in the *TP53*(−) cohort. In the future, a larger cohort should be collected to validate the phenomena discovered in this study.

Collectively, this study provides a novel perspective for the stratification of immunotherapy *via* mutational profiling in patients with NSCLC and suggests that *TP53* mutation status, as well as the biopsy lesion type, determines the difference in immunotherapy predictors.

## Data Availability Statement

The original contributions presented in the study are included in the article/supplementary material. Further inquiries can be directed to the corresponding authors.

## Author Contributions

Experiments were conceived and designed by BH, WZ, HW, and JL. Clinical analysis, bioinformatics analysis, and statistical analysis were performed by JL, RZ, YL, BZ, MH, YW, YC, ZY, and WZ. Figures and tables were generated by JL, RZ, and YL, and the manuscript was written by JL. The manuscript was revised by BH. All authors contributed to the article and approved the submitted version.

## Funding

This work was supported by the foundation of Shanghai Chest Hospital (Project Nos. 2019YNJCM11 and YJXT20190102); the Shanghai Leading Talents Program (2013), the Shanghai Jiao Tong University (Project Nos. 15ZH4009 and YG2021QN121); the key program of translational medicine from Shanghai Jiao Tong University School of Medicine (Project No. 15ZH1008); the foundation of Chinese Society of Clinical Oncology (Project Nos. Y-2019AZZD-0355 and Y-QL2019-0125); National Natural Science Foundation of China grants (Project No. 31801118).

## Conflict of Interest

The authors declare that the research was conducted in the absence of any commercial or financial relationships that could be construed as a potential conflict of interest.

## Publisher’s Note

All claims expressed in this article are solely those of the authors and do not necessarily represent those of their affiliated organizations, or those of the publisher, the editors and the reviewers. Any product that may be evaluated in this article, or claim that may be made by its manufacturer, is not guaranteed or endorsed by the publisher.
